# Loss of the Periplasmic Chaperone Skp and Mutations in the Efflux Pump AcrAB-TolC Play a Role in Acquired Resistance to Antimicrobial Peptides in *Salmonella typhimurium*

**DOI:** 10.3389/fmicb.2020.00189

**Published:** 2020-03-10

**Authors:** Gal Kapach, Reut Nuri, Christiane Schmidt, Adi Danin, Shir Ferrera, Alon Savidor, Roman G. Gerlach, Yechiel Shai

**Affiliations:** ^1^Departmant of Biomolecular Sciences, Weizmann Institute of Science, Rehovot, Israel; ^2^Project Group 5, Robert Koch Institute, Wernigerode, Germany; ^3^de Botton Institute for Protein Profiling, The Nancy and Stephen Grand Israel National Center for Personalized Medicine, Weizmann Institute of Science, Rehovot, Israel

**Keywords:** antimicrobial peptide (AMPs), bacterial resistance, AcrAB-TolC, ramR, Skp, periplasm, efflux pump

## Abstract

Bacterial resistance to antibiotics is a major concern worldwide, leading to an extensive search for alternative drugs. Promising candidates are antimicrobial peptides (AMPs), innate immunity molecules, shown to be highly efficient against multidrug resistant bacteria. Therefore, it is essential to study bacterial resistance mechanisms against them. For that purpose, we used experimental evolution, and isolated a *Salmonella enterica* serovar *typhimurium*-resistant line to the AMP 4DK5L7. This AMP displayed promising features including widespread activity against Gram-negative bacteria and protection from proteolytic degradation. However, the resistance that evolved in the isolated strain was particularly high. Whole genome sequencing revealed that five spontaneous mutations had evolved. Of these, three are novel in the context of acquired AMP resistance. Two mutations are related to the AcrAB-TolC multidrug efflux pump. One occurred in AcrB, the substrate-binding domain of the system, and the second in RamR, a transcriptional regulator of the system. Together, the mutations increased the minimal inhibitory concentration (MIC) by twofold toward this AMP. Moreover, the mutation in AcrB induced hypersusceptibility toward ampicillin and colistin. The last mutation occurred in Skp, a periplasmic chaperone that participates in the biogenesis of outer membrane proteins (OMPs). This mutation increased the MIC by twofold to 4DK5L7 and by fourfold to another AMP, seg5D. Proteomic analysis revealed that the mutation abolished Skp expression, reduced OMP abundance, and increased DegP levels. DegP, a protease that was reported to have an additional chaperone activity, escorts OMPs through the periplasm along with Skp, but is also associated with AMP resistance. In conclusion, our data demonstrate that both loss of Skp and manipulation of the AcrAB-TolC system are alternative strategies of AMP acquired resistance in *Salmonella typhimurium* and might represent a common mechanism in other Gram-negative bacteria.

## Introduction

Antimicrobial peptides (AMPs) are innate immunity molecules that exist in all life forms and act as the first line of defense against microorganisms. Antimicrobial peptides are mostly cationic, hydrophobic, and amphipathic, properties that facilitate their interaction and disruption of the polyanionic bacterial membranes (Shai, 2002; Zasloff, 2002). Accumulating evidence over the past two decades indicates that AMPs can be efficient against antibiotics-resistant bacteria (Lam et al., 2016; Mahlapuu et al., 2016; Chen et al., 2018; Lazar et al., 2018; Tucker et al., 2018). This makes them promising candidates as alternative antibiotics. Because AMPs might serve as future drugs and several AMPs are under clinical trials (Naafs, 2018), it is important to study bacterial resistance mechanisms against them.

Resistance to AMPs in Gram-negative bacteria is mediated mainly by surface modifications (Nuri et al., 2015), efflux pumps (Shafer et al., 1998), proteolytic degradation (Mattiuzzo et al., 2014), and biofilm formation (Kai-Larsen et al., 2010). *Salmonella enterica* serovar *typhimurium* is a model organism used to study resistance mechanisms toward AMPs. This bacterium has evolved to survive host environments containing AMPs within the small intestine and the phagosome (Gunn, 2008; Dalebroux and Miller, 2014). *In vivo* resistance to AMPs in *S. typhimurium* is known to be mediated by the two-component signal transduction systems (TCSs) PhoP-PhoQ (PhoPQ) or PmrA-PmrB (PmrAB). The PhoPQ TCS is activated in the presence of AMPs, low pH, or low Mg^2+^, whereas the PmrAB is induced by low pH, high Fe^3+^/Al^3+^, or by the PhoPQ system. Activation of both systems induces transcription of genes, eventually mediating lipopolysaccharide (LPS) modifications (Gunn, 2008), which are considered among the major resistance mechanisms against AMPs in *S. typhimurium*. These modifications mostly reduce the net negative surface charge, hence the electrostatic interactions between cationic AMPs and the bacterial anionic cell envelope (Matamouros and Miller, 2015; Nuri et al., 2015). Another strategy for LPS modification is lipid A palmitoylation by the PagP enzyme. This increases the hydrophobic interactions between the lipid A chains, thus altering the outer membrane fluidity, making it less permeable to AMPs (Guo et al., 1998; Bishop et al., 2000). Other reported resistance mechanisms involve the Sap efflux pump (Parra-Lopez et al., 1993) and proteolytic degradation by PgtE (Guina et al., 2000).

In the search for novel resistance mechanism of *S. typhimurium* to AMPs, we isolated a resistant line [resistant line 1 (RL1)] to the AMP 4DK5L7 by means of experimental evolution. This AMP is composed of five lysines and seven leucines, in which four of these amino acids were replaced by their D-enantiomers. We have shown previously that 4DK5L7 is highly potent against Gram-negative bacteria and non-hemolytic (Papo and Shai, 2005). Despite the promising properties of 4DK5L7, we found that the *S. typhimurium* isolate RL1 evolved high constitutive resistance toward this AMP. Genetic analysis revealed that RL1 carries five mutations in five loci. The first two mutations were found to be in the *yeiU* and the *rfaY* genes, which encode LPS biosynthesis enzymes. Specifically, *yeiU* and *rfaY* encode enzymes that add a phosphate group to the LPS lipid A (Touze et al., 2008) or to the LPS core (Heinrichs et al., 1998; Yethon et al., 1998), respectively. Loss of function or inhibition of these two genes was previously reported to confer resistance toward AMPs by reduction of the surface net negative charge (Herrera et al., 2010; Kato et al., 2012; Lofton et al., 2013). Interestingly, the other three mutations that we identified were never reported in the context of AMP acquired resistance. Two of the mutations are related to the AcrAB-TolC system, a multidrug resistance efflux pump that was mostly shown to expel antibiotics, bile salts, and dyes in Gram-negative bacteria (Yu et al., 2003; Pos, 2009). In *Escherichia coli* (*E. coli*) (Warner and Levy, 2010) and in *Klebsiella pneumoniae* (*K. pneumoniae*) (Padilla et al., 2009), deletion of AcrB, the substrate-binding domain of the system, caused susceptibility to AMPs. We found that RL1 carries a mutation within the genes for AcrB and RamR, a transcriptional repressor of AcrB expression (Abouzeed et al., 2008). The last mutation in RL1 is in a gene encoding the periplasmic chaperone Skp. This chaperone is involved in the biogenesis of outer membrane proteins (OMPs), escorting them through the periplasmic space toward the outer membrane (Sklar et al., 2007).

In this study, we evaluated the contribution of the mutations in AcrB, RamR, and Skp to AMP resistance. We found that introducing these mutations induced hyposensitivity toward the 4DK5L7 peptide. Moreover, the Skp mutation, which led to the loss of Skp, induced hyposensitivity to other AMPs as well. While Skp was mostly characterized in *E. coli* in the context of OMP biogenesis, we reveal that this gene plays a role in resistance to AMPs in *S. typhimurium* and hence might represent a common resistance mechanism in Gram-negative bacteria.

## Materials and Methods

### Peptide Synthesis and Purification

Peptides were synthesized by an automated peptide synthesizer liberty blue (CEM, Matthews, NC, United States) on rink amide 0.68 mmol/mg MBHA resin, using the Fmoc solid phase strategy. The resin-bound peptide was washed thoroughly with dimethylformamide and then dichloromethane, dried, and cleaved. Lipid groups were conjugated at this stage. Cleavage was done by addition of 95% trifluoroacetic acid (TFA), 2.5% water, and 2.5% triethylsilane. The crude peptides were purified by reverse-phase high-performance liquid chromatography. Purification of the peptides was done using a C4 or a C8 column (Grace Discovery Sciences, Columbia, MD, United States) in acetonitrile in water gradient (both containing 0.1% TFA) for 40 min.

### Bacterial Strains and Growth Condition

All *S. typhimurium* mutant strains used were derived from the ATCC 14028s background. Generally, cells were grown overnight in LB and before every experiment were regrown by a dilution of 1:100 into fresh sterile LB medium and incubated at 37°C with agitation for 2 h to reach a mid-log phase (OD_600 nm_ = 0.5–0.7), unless mentioned differently.

### Experimental Evolution and RL1 Isolation

The experimental selection assay was carried out in 96-well plates at a final volume of 100 μL as previously described (Lofton et al., 2013) with several alterations. The 4DK5L7 peptide was dissolved in double distilled water (DDW) to the appropriate concentration, and 5 μL of the peptide was added to 45 μL of Mueller-Hinton broth (MHB) in a flat-bottom 96-well plate (four replicates). Five microliters of DDW was used only for the wild type (WT) as a negative control. Fifty microliters of WT *S. typhimurium* culture (approximately 10^5^ bacteria/mL) in MHB was added to the peptide-containing 96-well plate. The plate was incubated at 37°C with shaking for approximately 24 h. Every day, 10 μL of bacteria from each lineage was transferred to 90 μL of a fresh medium containing peptides, in a new 96-well plate. After transfer, 10 μL of 70% glycerol was added to each culture in the old plate, and the 96-well plates were kept at −80°C. If the bacteria in a well did not grow after transfer, the lineage was restarted from the most recent passage that showed successful growth. When a lineage survived three subsequent transfers, the peptide concentration was increased by 50% for approximately 30 passages. RL1 was isolated from the well in which the maximal concentration of a peptide was achieved. Bacteria were spread on LB agar plate, and a single colony was collected. The colony was grown in liquid LB for three passages without a peptide, and then the minimal inhibitory concentration (MIC) was determined.

### MIC Determination

Mid-log cultures (OD_600_ = 0.5–0.7) were adjusted to final OD_600_ of 0.005 in MHB. Peptides or antibiotics were dissolved in DDW and added to the MHB for the appropriate final concentration. Plates were incubated for 18 h at 37°C with agitation. Inhibition of growth was determined by absorbance measurements at 600 nm using a microplate autoreader. Alternatively, absorbance at 600 nm was measured at 37°C with agitation, every 20 min for 18 h in Cytation 5 plate reader (BioTek, Winooski, VT, United States) to evaluate the growth curves. Growth rates were calculated using the R package “growth rates” according to Kahm et al. (2010). Data were filtered for growth (OD_600_ > 0.2), and growth rates for data sets not reaching that threshold were set to zero.

### Whole Genome Sequencing

DNA was purified from the control strain and RL1 overnight cultures using the DNeasy Blood & Tissue Kits (Qiagen, Germany). Sequencing was performed on an Illumina MiSeq platform, as previously described (Blecher-Gonen et al., 2013) with the following modifications: 300 to 600 ng of gDNA was sheared using the Covaris E220X sonicator (Covaris, Inc., Woburn, MA, United States). End repair was performed in 80-μL reaction at 20°C for 30 min, followed by Agencourt AmPURE XP beads cleanup (Beckman Coulter, Inc., Indianapolis, IN, United States) in a ratio of 0.75 × beads/DNA volume. A-bases were added to both 3′ ends, followed by adapter ligation in a final concentration of 0.125 μM. An SPRI bead cleanup in a ratio of 0.75 × beads/DNA volume was performed, followed by eight polymerase chain reaction (PCR) cycles using 2 × KAPA HiFi ready mix (Kappa Biosystems, Inc., Wilmington, MA, United States) in a total volume of 25 μL with the following program: 2 min at 98°C, eight cycles of 20 s at 98°C, 30 s at 55°C, and 60 s at 72°C followed by 72°C at 10 min. Sequencing QC was performed using fastqc website. Reads were mapped to *S. typhimurium* strain 14028s genome version ASM2216v1 using BWA-MEM v0.7.12-r1039 (Li and Durbin, 2009), sorted using SAMtools v1.2 website. Polymerase chain reaction duplicate reads were identified and marked using Picard MarkDuplicates. GATK HaplotypeCaller v3.5 (McKenna et al., 2010) was used to discover single-nucleotide polymorphisms (SNPs) and insertions/deletions, using the setting-ploidy 10 in order to increase sensitivity to subclonal variants. The resulting variants were hard filtered using standard GATK filters for base quality, strand bias, and so on. Variant allele fractions for each variant and each sample were computed from the allelic depths field in the VCF. Variants were annotated for predicted effect using snpEFF (Cingolani et al., 2012).

### RNA Isolation and Quantitative Reverse Transcriptase-PCR

RNA was purified from cells at the mid-log stage. Cells were centrifuged at 4,000 revolutions/min for 10 min. The pellet was resuspended with 150 μL of fresh 2 mg/mL lysozyme (Sigma, Germany), Tris 1M, EDTA 0.5M, pH 8.0. Following incubation of 3 to 5 min at 37°C, 1 mL of Tri-reagent (Sigma, Germany) was added. The mixture was vortexed for 10 s at maximum speed and then incubated at room temperature for 5 min. After addition of 200 μL chloroform, the mixture was vortexed until homogeneity. The mixture was incubated at room temperature for 5 min until phase separation has appeared and subsequently centrifuged at 12,000 *g* for 15 min at 4°C. The upper phase was collected into a new tube, and 500 μL of isopropanol was added. The tube was inverted gently and incubated at room temperature for 10 min and then at −20°C for 30 min. The mixture was centrifuged at maximum speed for 25 min. The pellet was washed twice with 70% ethanol and left to dry. The pellet was resuspended in 30 μL of DDW and incubated at 55°C for 10 min until completely dissolved. One microgram of the RNA was reverse transcribed using Superscript II reverse transcriptase (Invitrogen, Carlsbad, CA, United States), dNTPs, and random hexamers. Quantitative reverse transcriptase (qRT)–PCR was performed in 7300 Real time PCR system (Applied Biosystems) with Platinum SYBR Green qPCR Super Mix-UDG with ROX (Invitrogen). The qRT-PCR was performed in duplicates, three biological replicates in one plate. The values were normalized to the *gyrB* gene.

### Reconstruction of the Mutated Genes in the Control Strain and HA Epitope Tagging of AcrB

Point mutations and HA epitope tagging in the bacterial genome were performed using the pWRG730 and pWRG717 constructs, as described previously (Hoffmann et al., 2017). Briefly, the ISceI + kanamycin cassette was amplified from the pWRG717 plasmid with primers containing homology parts to the target gene (see primers information in Supplementary Table S1). *Salmonella typhimurium* carrying the pWRG730 were grown to an OD_600_ = 0.4–0.5, and λ red recombinase expression was induced by incubation at 42°C for 12.5 min and then transferred to ice for 15 min. The cells were prepared for electroporation by three washes in 10% ice-cold glycerol. The pellet was resuspended in 100 μL of 10% glycerol, and 50 to 100 ng of the PCR product was added. The cells were electroporated and were recovered in LB at 30°C for 1 h. Then, they were selected on kanamycin plates, and positive clones for homologous recombination were chosen. A 300-bp fragment was amplified from RL1 genome, designed to contain the point mutation or the epitope tag. Expression of the λ red recombinase was induced in the positive clones as described above. Next, the clones were electroporated with the 300-bp fragment containing the point mutation or the HA epitope tag. Cells were selected on chloramphenicol plates containing 100 ng/mL anhydrotetracycline. Positive clones were verified by colony PCR and sequencing.

### Western Blot Analysis

Cells were grown to mid-log stage, and OD_600 nm_ was checked. For OD_600 nm_ of less than 0.5, 1 mL of culture was collected; for 0.5 < OD_600 nm_ < 1, 0.5 mL of culture was collected, and for OD_600 nm_ > 1, 0.25 mL was collected. Cells were centrifuged at 6,000 *g* for 10 min, at 4°C. The supernatant was removed, and the pellet was resuspended in 1 × sample buffer (Tivan-Biotech, Kfar Sava, Israel) according to the following formula: sample buffer vol (mL) = 0.5 × vol sample × OD_600 nm_. The samples were heated to 95°C for 10 min, cooled down on ice, and were loaded on 12% sodium dodecyl sulfate-polyacrylamide gel electrophoresis gel, 110 V. Proteins were transferred to a polyvinylidene difluoride membrane using wet transfer overnight at 30 V. The blots were blocked for 1 h in TBST + 5% milk, washed with TBST, and incubated overnight at 4°C with an anti-HA antibody (C29F4) rabbit Mab #3724 (Cell Signaling Technology, Danvers, MA, United States). The blots were washed three times with TBST and incubated for 1 h with goat anti-rabbit horseradish peroxidase-conjugated secondary antibody (Jackson Immuno Research Laboratories, Inc., West Grove, PA, United States) The treated blots were developed on an enhanced chemiluminescence detection system with Luminata^TM^ Crescendo Western HRP substrate (Merck Millipore, Burlington, MA, United States). For the housekeeping gene, the blots were incubated with anti GroEL antibody produced in rabbit (Sigma). Quantification of bands intensity was performed in ImageJ software^1^.

### Proteomic Analysis

Cells were grown to mid-log phase, centrifuged at 4,000 *g* for 5 min, and pellet was frozen on dried ice. The samples were subjected to lysis and in solution tryptic digestion using the S-Trap method [by ProtiFi (Huntington, New York, NY, United States)]. The resulting peptides were analyzed using nanoflow liquid chromatography (nanoAcquity) coupled to high-resolution, high-mass-accuracy mass spectrometry (Q-Exactive HFX). Each sample was analyzed on the instrument separately in a random order in discovery mode. Raw data were processed with MaxQuant (Cox and Mann, 2008). The data were searched with the Andromeda search engine against the *S. typhimurium* 14028s proteome database appended with common laboratory protein contaminants and the following modifications: fixed modification—cysteine carbamidomethylation; variable modifications—methionine oxidation, asparagine, and glutamine deamidation. For the single-gene analysis, the quantitative comparisons were calculated using Perseus (Tyanova et al., 2016). Decoy hits were filtered out. Only proteins that had at least two valid values in at least one experimental group were kept. Pathway enrichment analyses were done on the basis of the current Kyoto Encyclopedia of Genes and Genomes (KEGG) annotation of the *S. typhimurium* ATCC 14028s genome (“seo”) using the package “clusterProfiler” (Yu et al., 2012) in “R” v3.51^2^.

## Results

### RL1 Isolation and Characterization

RL1 was isolated in our laboratory using experimental evolution. The WT *S. typhimurium*, with a MIC of 12.5 μM (Table 1) were exposed to a sub-inhibitory concentration (0.5 × MIC) of the 4DK5L7 peptide. Following three successful passages, we increased the concentration by 50%. In total, 30 passages were conducted, and the maximal concentration in which bacteria exhibited growth was 160 μM (12.8 × MIC) (Table 1). As a control, we passaged the WT bacteria with DDW at the equivalent volume to the peptide for the same amount of passages. From the last passage, we isolated a single colony, which was then grown in the absence of the peptide for 3 days. The isolate that was passaged with 4DK5L7 was named RL1, and the isolate that was passaged with DDW instead of the peptide and will be used in our further experiments was named “control strain.” Next, we tested the MIC of the control strain and RL1 for the 4DK5L7 peptide and to other AMPs: seg5D, C8-K5L7, and colistin (Table 2). We found that RL1 exhibited a high resistance level toward 4DK5L7, as the MIC was 200 μM compared to 12.5 μM in the control strain (Table 1). Moreover, RL1 showed cross-resistance toward another AMP, seg5D, a synthetic AMP that was designed in our laboratory (Papo et al., 2002). RL1 showed a MIC of 50 μM compared to 6.25 to 12.5 μM in the control strain (Table 1). RL1 showed hyposensitivity toward the cyclic lipopeptide colistin of the polymyxin family and to the synthetic lipopeptide C8-K5L7. RL1’s MIC to colistin was detected as 6.25 versus 1.56 to 3.125 μM in the control strain and to C8-K5L7 6.25 to 12.5 versus 6.25 μM in the control strain (Table 1).

**TABLE 1:** Bacterial strains used in this study and their MICs.

Bacterial strain	Relevant characteristics/genotype^a,b,c^	Source	4DK5L7 MIC (μM)	seg5D MIC (μM)	Colistin MIC (μM)	C8-K5L7 MIC (μM)
WT	*S. typhimurium* 14028s	ATCC	6.25–12.5	6.25–125	3.125	6.25
Control strain	*skp*^WT^, *ramR*^WT^, *acrb*^WT^, *yeiU*^WT^, *rfaY*^WT^	This study	12.5	6.25–12.5	1.56–3.125	6.25
RL1	*skp*, *ramR*, *acrb*, *yeiU*, *rfaY*	This study	200	50	6.25	6.25–12.5
*acrB* mut	*acrB*	This study	12.5	6.25	ND	ND
Double mutant	*acrB*, *ramR*	This study	25	6.25–12.5	1.56	6.25
*ramR* mut	*ramR*	This study	12.5	6.25	ND	ND
*skp mut*	*Skp*	This study	25	50	3.125	6.25
Triple mutant	*skp*, *ramR*, *acrB*	This study	50	50	3.125	6.25
Control strain HA	*acrb*^WT^-HA	This study	12.5	ND	ND	ND
RL1 HA	*skp*, *ramR*, *acrb*-HA, *yeiU*, *rfaY*	This study	>50	ND	ND	ND
*acrB* mut HA	*acrb*-HA	This study	12.5	ND	ND	ND
Double mutant HA	*ramR*, *acrb*-HA	This study	25	ND	ND	ND
*ramR* mut HA	*ramR*, *acrb*^WT^-HA	This study	12.5	ND	ND	ND

*^*a*^Italics indicate on a point mutation in the mentioned gene. ^*b*^*skp*^*WT*^, *ramR*^*WT*^, *acrb*^*WT*^, *yeiU*^*WT*^, *rfaY*^*WT*^ indicate on a WT allele. ^*c*^HA indicates on an HA epitope tag that was attached to the C terminus of the gene. ND indicates not determined.*

**TABLE 2:** Peptides used in the study.

Peptide	Sequence^a^
4DK5L7	KLLLKLKLKLLK- NH_2_
Seg5D	KKKLLLLLLLLLKKK-NH2
Colistin	C_52_H_98_N_16_O_13_
C8-K5L7^b^	C8-KLLLKLKLKLLK- NH_2_

*^*a*^Underlined amino acids refer to D enantiomers. ^*b*^This lipopeptide is composed of an eight-carbon fatty acid (caprylic acid) attached to the K5L7 peptide.*

### Whole Genome Sequencing of RL1 Reveals Five Mutations

**FIGURE 1:**
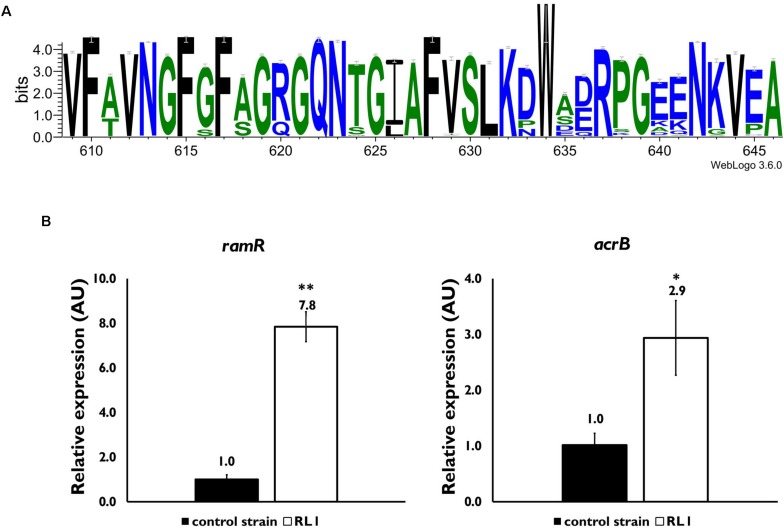
Characterization of the effect of mutations in *acrB* and *ramR.* **(A)** A consensus sequence logo of the switch loop was generated through https://weblogo.berkeley.edu/ using the multiple sequence alignment tool CLUSTAL 2.1. Sequences of 998 Gram-negative bacterial species and strains were used. **(B)** Quantitative reverse transcriptase-polymerase chain reaction (PCR) analysis of the relative expression of the *ramR* (left panel) and *acrB* (right panel) mRNA levels, in mid-log phase bacteria. The values are average of three biological replicates, each performed in triplicates. Statistical analysis was done using *t*-test. ^∗^*P* < 0.05, ^∗∗^*P* < 0.01.

In order to investigate the mutations that were acquired in RL1, DNA was purified from the control strain and RL1 bacteria, and whole genome sequencing was performed. While no mutations occurred in the control strain, RL1 carried five mutations in five loci: *hlpA* (*skp*), *acrB*, *ramR*, *yeiU*, and *rfaY* (Table 3). *hlpA* (histone-like protein A) encodes the Skp chaperone that facilitates the translocation of membrane proteins from the inner to the outer membrane, preventing protein aggregation in the periplasmic space (Schafer et al., 1999). RL1 carried a deletion mutation in position 265,722, resulting in a frameshift that eventually led to a premature stop codon within *hlpA* (Table 3). The *acrB* gene encodes AcrB, the substrate-binding domain of the AcrAB-TolC system, a multidrug efflux pump. This system is mostly known to protect bacteria from bile salts, dyes, and antibiotics such chloramphenicol and tetracycline (Adhya et al., 1995; Yu et al., 2003). There is evidence that several AMPs are substrates of the system. Two studies reported that deletion of this system increased *K. pneumoniae* and *E. coli* susceptibility to AMPs, respectively (Padilla et al., 2009; Warner and Levy, 2010). The AcrB mutation in RL1 is located at arginine 620, which was replaced by serine (R620S). Based on previous structural studies, this amino acid is located within a switch loop that allows the conformational change of AcrB monomers and is important for substrate access and binding (Eicher et al., 2012). We compared the conservation of the switch loop sequence among ∼1,000 Gram-negative bacterial strains, which include ∼240 different species. We found that, generally, this domain is highly conserved (Figure 1A). The R/Q620 position is well-conserved and might indicate a functional importance (Figure 1A). *ramR* encodes a transcriptional regulator which activity was previously described in *S. typhimurium* LT2. Accordingly, RamR represses the transcription of *ramA*, an activator of the *acrA* and *acrB* expression (Abouzeed et al., 2008). In RL1, the mutation is located at position 639,370, which led to substitution of threonine 19 with proline (T19P). This amino acid is located in the alpha helix α1, a part of the protein DNA-binding domain (Yamasaki et al., 2013). Because proline is known to disrupt alpha helical structures (Li et al., 1996), it could potentially affect RamR’s ability to bind the *ramA* promoter. Quantification of *ramR* transcripts indicated a 7.8-fold increase in RL1 compared to the control strain (Figure 1B). Yet, the *acrB* transcript levels were higher in RL1 than in the control strain (Figure 1B), implying that the repressive activity of RamR might be impaired in RL1.

**TABLE 3:** Description of RL1’s mutations.

Gene	Position	Ref^a^	Alt^b^	Type^c^	Effect^d^
*hlpA (skp)*	265722	AC	A	DEL	Frameshift
*acrB*	531233	G	T	SNP	Missense
*ramR*	639370	T	G	SNP	Missense
*yeiU*	2364176	G	A	SNP	Stop codon
*rfaY*	3925953	AT	A	DEL	Frameshift

*^*a*^The nucleotide at the position in the reference genome of *Salmonella enterica* serovar *typhimurium* 14028s. ^*b*^The nucleotide at the position in RL1 genome. ^*c*^The type of mutation: DEL, deletion; SNP, single-nucleotide polymorphism. ^*d*^The predicted effect of the mutation on the protein.*

The gene products of *yeiU* and *rfaY* are involved in LPS biosynthesis. *yeiU* encodes the inner membrane protein LpxT, which transfers a phosphate group to lipid A, forming the 1-diphosphate species (Touze et al., 2008). RL1 carries an SNP mutation in *yeiU*, which resulted in a stop codon (Table 3). Inactivation of this protein was shown to confer resistance to AMPs due to reduction of the net negative surface charge (Herrera et al., 2010; Kato et al., 2012). *rfaY* encodes WaaY, which is involved in phosphorylation of the HepII of the LPS core (Yethon et al., 1998). RL1 carries a deletion mutation in this locus, which resulted in a frameshift effect (Table 3). A frameshift mutation in this gene was previously reported to evolve in experimental evolution that induced AMP resistance. It was suggested that the reduction in the LPS net negative charge weakens the interaction with positively charged AMPs as a mechanism of resistance (Lofton et al., 2013). Because mutations with similar impact both in *yeiU* and *rfaY* were already reported to confer resistance to AMPs, we focused on the mutations in *acrB*, *ramR*, and *skp* and their possible contribution to AMP resistance.

### The Combination of Mutations in AcrB and RamR Induces Hyposensitivity Toward 4DK5L7 but Not to seg5D

**FIGURE 2:**
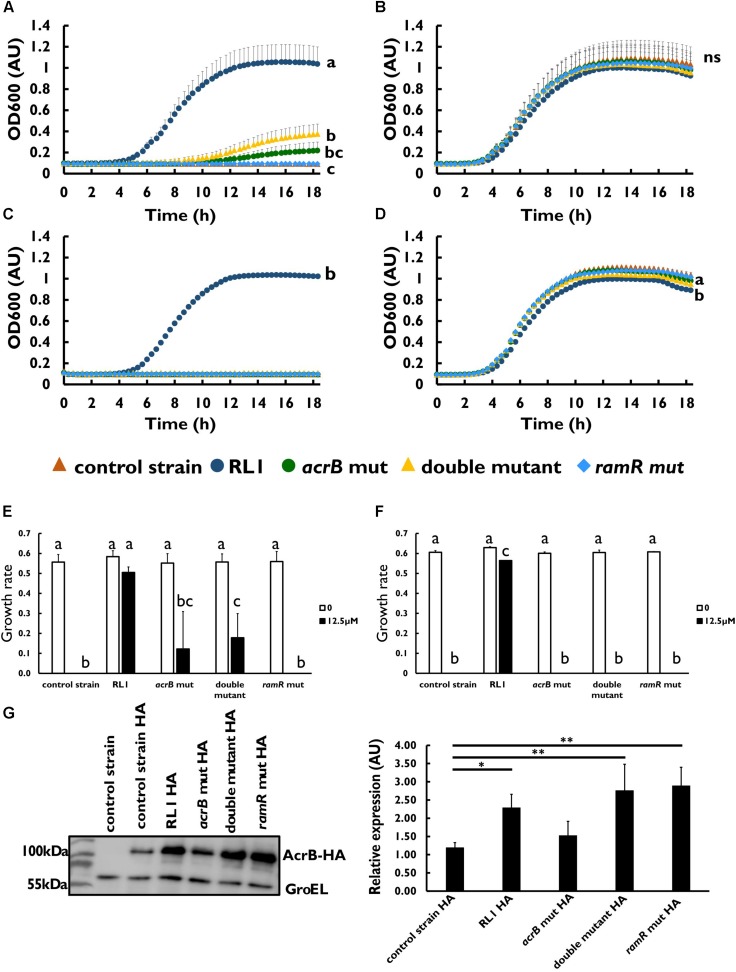
The effect of mutations in *acrB* and *ramR* on resistance and on AcrB protein levels. Growth curve of bacterial strains at 12.5 μM of **(A)** 4DK5L7 peptide and **(B)** without peptide. Growth curve of bacterial strains at **(C)** 12.5 μM seg5D peptide or **(D)** without peptide. Growth rates of the strains at the different peptide concentrations of **(E)** 4DK5L7 and **(F)** seg5D. OD600 was monitored every 20 min for 18 h. Statistical analysis was performed using Tukey multiple comparison test for all the time points. Statistically significant differences are indicated as a≠b≠c, *P* < 0.05. The values are average of at least three biological replicates, each performed in duplicates. Error bars indicate the standard error. **(G)** Left panel: acrB-HA protein levels in mid log phase bacteria were measured by western blot. The control strain bacteria without the HA epitope tag was used as a negative control. Anti-GroEL antibody was used for loading control. Right panel: quantification of the AcrB-HA relative expression, an average of three biological replicates. Statistical analysis was performed using Dunette multiple comparison analysis. ^∗^*P* < 0.05, ^∗∗^*P* < 0.01.

To investigate whether the mutations in AcrB and RamR contribute to resistance toward the 4DK5L7 peptide, we generated mutants carrying RL1’s point mutations in *acrB* (*acrB* mut), in *ramR* (*ramR* mut), and both of these mutations (double mutant) (Table 3). Next, we compared the growth of the control strain, RL1, *acrB* mut, double mutant, and *ramR* mut in the presence of 4DK5L7 at the MIC of the control strain (12.5 μM). As expected, RL1 showed a remarkable optimal growth, whereas the control strain did not exhibit growth (Figure 2A). The double mutant, having a MIC twofold higher than the control strain (Table 1), showed a moderate growth, which was significantly higher than the control strain but lower than RL1 (Figure 2A). The *acrB* mut and the *ramR* mut bacteria displayed a similar MIC to the control strain (Table 1), and no significant differences between their growth curves were observed (Figure 2A). As a negative control, bacteria were grown without peptide and showed no significant differences in growth (Figure 2B). Furthermore, we checked whether the mutations contribute to resistance against seg5D, a peptide that RL1 showed cross-resistance toward (Table 1). The MIC of the *acrB* mut, *ramR* mut, and the double mutant was similar to that of the control strain (Table 1). Accordingly, at the MIC of the control strain (12.5 μM), RL1 exhibited an optimal growth, whereas none of the mutants grew (Figure 2C). In the negative control, the strains showed similar growth, whereas RL1 reached a slightly lower OD_600_ value (Figure 2D). Based on the growth curves, we calculated the growth rates of the strains in the presence of these AMPs. We found that at 12.5 μM of 4DK5L7 the double mutant and the *acrB* mut had similar low growth rates. However, the double mutant was significantly different from the control strain, whereas the *acrB* mut was not (Figure 2E). In addition, RL1’s growth rate was only slightly reduced at 12.5 μM of 4DK5L7 (Figure 2E) and 12.5 μM of seg5D (Figure 2F) compared to the growth rate in the absence of AMPs.

### The AcrB Mutant Protein Is Overexpressed in RL1 and the Double Mutant

Next, we aimed to validate the protein expression levels of the mutated AcrB and to test the impact of the mutation in *ramR* on the protein level. For this purpose, an epitope tag was added to the C-terminal end of the protein (Table 1). The addition of an HA epitope tag to the AcrB protein did not affect the MIC toward 4DK5L7 among the mutants and the control strain (Table 1 and Supplementary Figure S1). Using Western blot analysis, we found that the *acrB* mut and the control strain bacteria had similar AcrB-HA protein levels (Figure 2G). Bacteria carrying the point mutation in the *ramR* gene (RL1, double mutant, and *ramR* mut) showed a significant higher levels of AcrB than the control strain (a fold change of 2.2–2.9) (Figure 2G). This fold change is similar to the elevation in the *acrB* transcripts that were measured in RL1 (Figure 1B).

### The Mutation in RamR Induced Hyposensitivity Toward Chloramphenicol and Ampicillin While the Mutation in AcrB Induced Hypersusceptibility Toward Ampicillin

**FIGURE 3:**
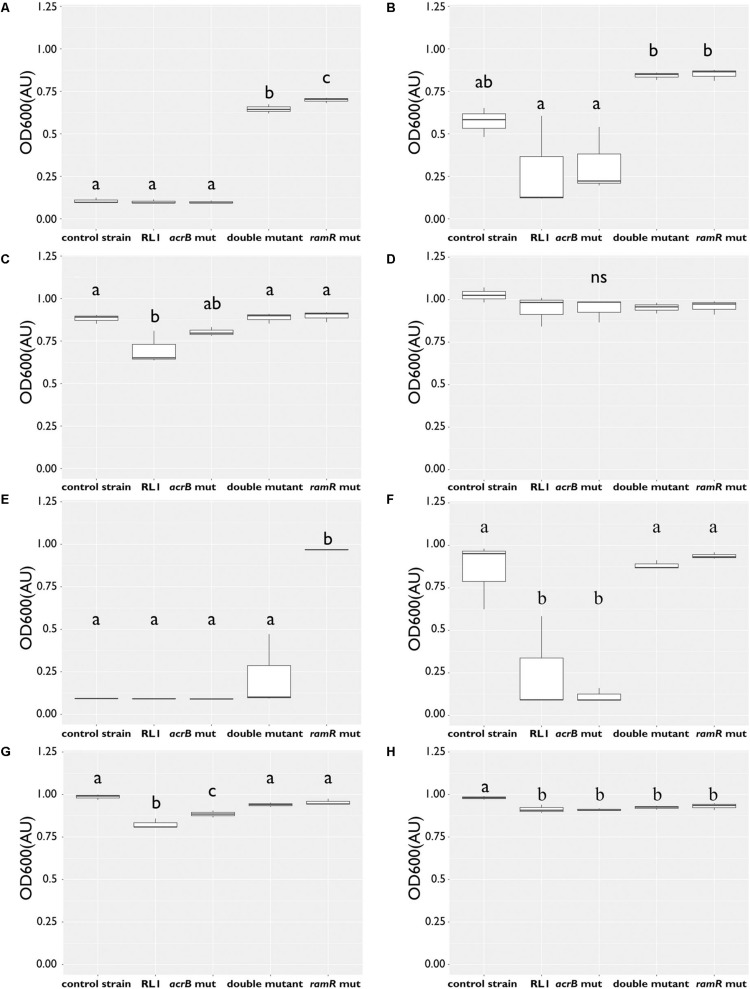
The mutation in *ramR* induced hyposensitivity to chloramphenicol and ampicillin, whereas the mutation in *acrB* induced hypersusceptibility to ampicillin. Bacteria were grown overnight with antibiotics at 37°C to determine their minimal inhibitory concentration (MIC) to chloramphenicol or ampicillin. OD_600_ of the control strain RL1, *acrB* mut, double mutant, and *ramR* mut was measured following 18 h. OD_600_ of bacteria grown with chloramphenicol at concentration of **(A)** 5.8 μM, **(B)** 2.9 μM, **(C)** 1.8 μM, and **(D)** at the absence of chloramphenicol. OD_600_ of bacteria grown with ampicillin at concentration of **(E)** 33.6 μM, **(F)** 16.8 μM, **(G)** 8.4 μM, and **(H)** at the absence of ampicillin. The values are average of three biological replicates; each was performed in duplicates. Statistical analysis was performed using Tukey multiple-comparisons test. Statistically significant differences are indicated as a≠b≠c, *P* < 0.05.

Because the AcrAB-TolC system was mostly studied in the context of resistance to antibiotics, we evaluated the impact of RL1’s mutations on the MIC of chloramphenicol and ampicillin compared to the control strain. We found that the mutation in *ramR* increased the MIC of chloramphenicol and ampicillin, whereas the mutation in *acrB* reduced the MIC of ampicillin (Table 4). More specifically, at 5.8 μM (1 × MIC of the control strain), no growth was observed for the control strain, RL1 and *acrB* mut. This is in contrast to the double mutant and the *ramR* mut (Figure 3A). At 2.9 μM (0.5 × MIC of the control strain), RL1 and the *acrB* mut were hyposensitive to chloramphenicol compared to the other strains (Figure 3B). At 1.45 μM (0.25 × MIC of the control strain), all the strains grew, while RL1 was yet hyposensitive to chloramphenicol than the others (Figure 3C). No significant differences in growth were observed in the absence of chloramphenicol (Figure 3D). In the case of ampicillin, we found more significant impact of the mutation in *acrB* on the MIC values (Table 4). At 33.6 μM (1 × MIC of the control strain), *ramR* mut was the only strain that exhibited growth (Figure 3E). At 16.8 μM (0.5 × MIC of the control strain), the control strain, double mutant, and *ramR* mut exhibited growth, whereas RL1 and *acrB* mut did not (Figure 3F). At 8.4 μM (0.25 × MIC of the control strain), all the strains showed growth, whereas RL1 and *acrB* mut were slightly more susceptible (Figure 3G). In the absence of ampicillin, all strains grew similarly, whereas the control strain exhibited a minor advantage (**Figure 3H**).

**TABLE 4:** MIC to antibiotics.

	Control	RL1	*acrB*	Double	*ramR*
	strain	RL1	mut	mutant	mut
Chloramphenicol (μM)	5.8	2.9–5.8	2.9–5.8	11.6	11.6
Ampicillin (μM)	33.6	16.8–33.6	16.8	33.6	67.2

*The values are representative data from three biological replicates, each performed in duplicates.*

### The Mutation in *skp* Induced Hyposensitivity to 4DK5L7 and Cross Resistance Toward seg5D

**FIGURE 4:**
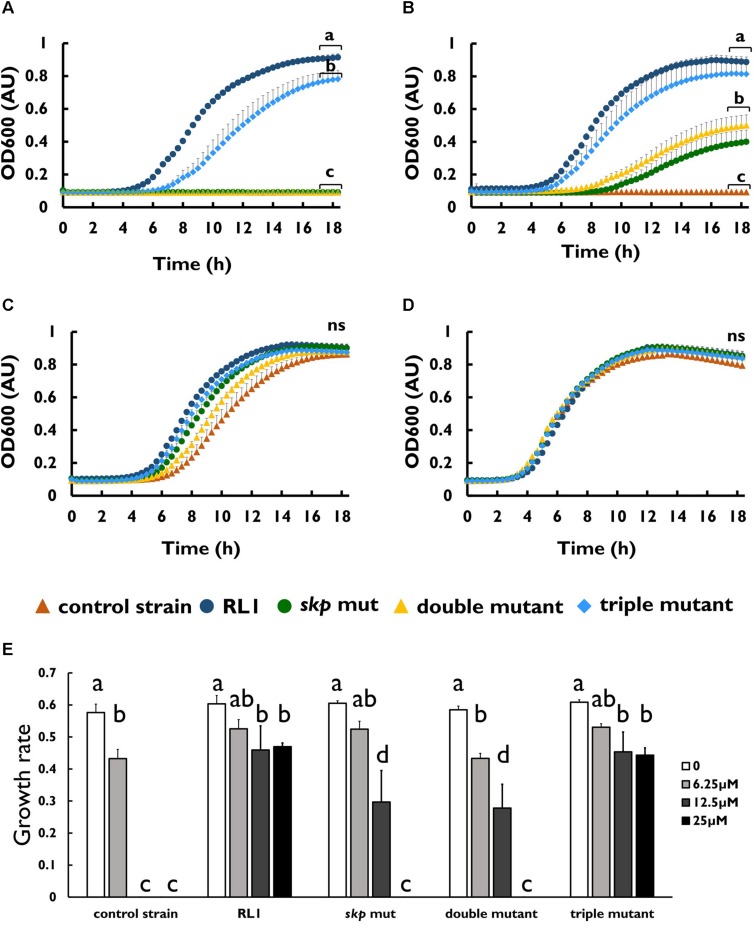
The mutation in *skp-*induced hyposensitivity to the 4DK5L7 peptide. Growth curves of bacterial strains with the 4DK5L7 peptide at **(A)** 25 μM, **(B)** 12.5 μM, **(C)** 6.25 μM, and **(D)** without peptide. **(E)** Growth rates of the strains at the different peptide concentrations. OD_600_ was monitored every 20 min for 18 h. The values are average of at least three biological replicates, each performed in duplicates. Error bars indicate the standard error. Statistical analysis was performed using Tukey multiple-comparisons test for the last five the time points. Statistically significant differences are indicated as a≠b≠c≠d, *P* < 0.05.

**FIGURE 5:**
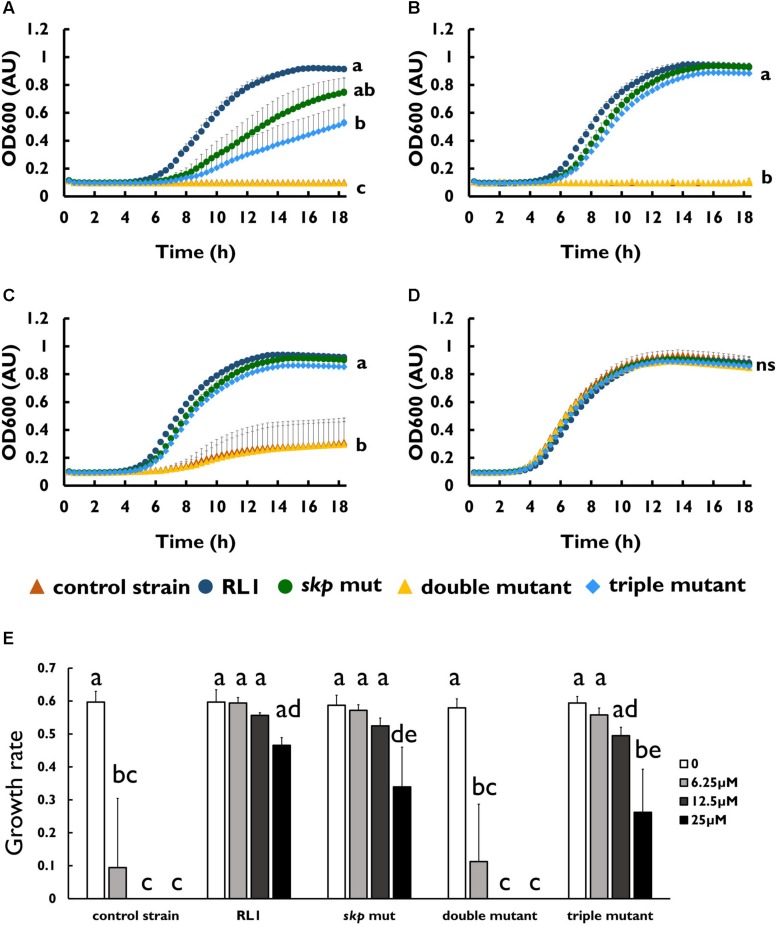
*skp* mut but not the double mutant is hyposensitive to the seg5D peptide. Growth curves of bacterial strains with the seg5D peptide at **(A)** 25 μM, **(B)** 12.5 μM, **(C)** 6.25 μM, and **(D**) without peptide. **(E)** Growth rates of the strains at the different peptide concentrations. OD_600_ was monitored every 20 min for 18 h. The values are average of at least three biological replicates, each performed in duplicates. Error bars indicate the standard error. Statistical analysis was performed using Tukey multiple-comparisons test for all the time points. Statistically significant differences are indicated as a≠b≠c≠d≠e, *P* < 0.05.

**FIGURE 6:**
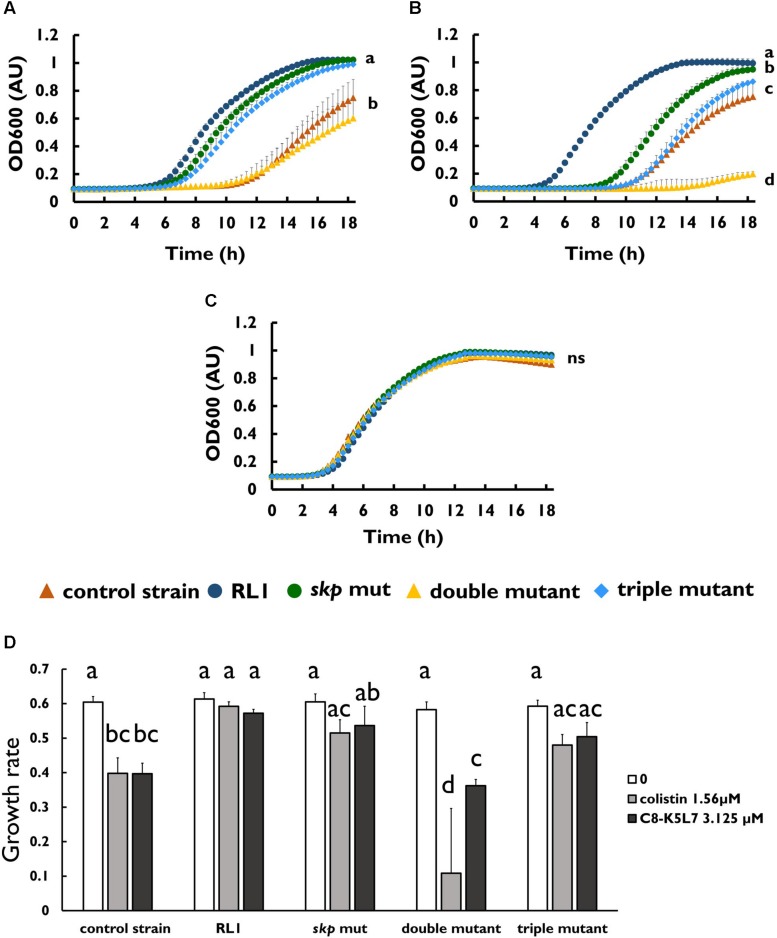
Mutation in *skp* improved the growth in sub-minimal inhibitory concentration concentrations of C8-K5L7, whereas double mutant was hypersusceptible to colistin. Growth curves of bacterial strains **(A)** with C8-K5L7 at 3.125 μM, **(B)** with colistin at 1.56 μM, or **(C)** without peptide. **(D)** Growth rates of the strains at the different peptide concentrations. OD_600_ was monitored every 20 min for 18 h. The values are average of at least three biological replicates, each performed in duplicates. Error bars indicate the standard error. Statistical analysis was performed using Tukey multiple-comparisons test for all the time points. Statistically significant differences are indicated as a≠b≠c≠d, *P* < 0.05.

To investigate whether the mutation in *skp* contributes to resistance to AMPs, we introduced this point mutation in the control strain background (*skp* mut) and in the double-mutant background (triple mutant) (Table 1). We found that the MIC to 4DK5L7 was increased by twofold in the *skp* mut and by fourfold in the triple mutant. A comparison between the growth curves of the different strains revealed that at 25 μM (2 × MIC of the control strain) RL1 showed an optimal growth, and the triple mutant showed a suboptimal growth (Figure 4A). The other bacterial strains did not exhibit any growth at this concentration (Figure 4A). At 12.5 μM (1 × MIC of the control strain), RL1 and the triple mutant showed a similar optimal growth (Figure 4B). The *skp* mut and the double mutant showed a similar moderate growth, whereas the control strain did not grow (Figure 4B). At 6.25 μM (0.5 × MIC of the control strain) and without peptide, no significant differences were observed between the five strains (Figures 4C,D, respectively). The growth rates of RL1 and the triple mutants were similar at the different peptide concentrations (Figure 4E). The *skp* mut and the double mutant exhibited similar growth rates that were lower compared to RL1 and the triple mutant at 12.5 μM (Figure 4E). Next, we tested the impact of the mutation in *skp* on the MIC of the seg5D peptide. We found that the MIC of the *skp* mut and the triple mutant increased by fourfold (Table 3). Comparison between the growth curves of the strains demonstrated that the *skp* mut and the triple mutant showed a moderate growth at 25 μM (2 × MIC of the control strain) (Figure 5A). RL1 showed an optimal growth, whereas the control strain and the double mutant did not grow (Figure 5A). At 12.5 μM (1 × MIC of the control strain) RL1, *skp* mut and the triple mutant showed similar optimal growth, whereas the control strain and the double mutant did not exhibit growth (Figure 5B). At 6.25 μM (0.5 × MIC of the control strain) RL1, *skp* mut and the triple mutant reached higher OD_600_ levels compared to the control strain and the double mutant (Figure 5C). No significant differences between the growth curves of the five strains were observed without peptide (Figure 5D). The growth rates at the different concentrations of seg5D were similar between the control strain and the double mutant and between the *skp* mut and the triple mutant (Figure 5E). The growth rates of RL1 were mostly similar to the *skp* mut and the triple mutant except of the growth rate at 25 μM, which was higher for RL1 (Figure 5E). We further tested the growth in the presence of other AMPs and observed variations in growth at sub-inhibitory concentrations. In the presence of 3.125 μM C8-K5L7 peptide (0.5 × MIC of the control strain) RL1, *skp* mut and the triple mutant had growth advantages due to a shorter lag phase and higher maximal OD_600_ values compared to the control strain and the double mutant (Figure 6A). We also found differences in growth in the presence of 1.56 μM colistin (0.5 × MIC of the control strain). RL1 showed an optimal growth, and *skp* mut showed a slightly slower growth than RL1 (Figure 6B). The triple mutant and the control strain showed similar growth (Figure 6B). The double mutant showed a negligible growth under these conditions, suggesting colistin hypersusceptibility of that strain (Figure 6B). Once more, no differences were observed between the strains in the absence of peptides (Figure 6C). The growth rates of the control strain were significantly reduced at 1.56 μM of colistin or 3.125 μM of C8-K5L7, opposed to RL1, *skp* mut, and the triple mutant (Figure 6D). Moreover, the growth rate of the double mutant was significantly reduced at 1.56 μM of colistin compared to all the other strains (Figure 6D). Overall, the *skp* mutation induced hyposensitivity toward 4DK5L7 and C8-K5L7 and resistance toward seg5D. The double mutant showed hyposensitivity toward 4DK5L7 and hypersusceptibility to colistin. A combination of the three mutations in *ramR*, *acrB*, and *skp* resulted in a significant resistance to the 4DK5L7 peptide.

### The Mutation in *skp* Led to the Loss of the Skp Protein and Influenced Multiple Cellular Pathways

**FIGURE 7:**
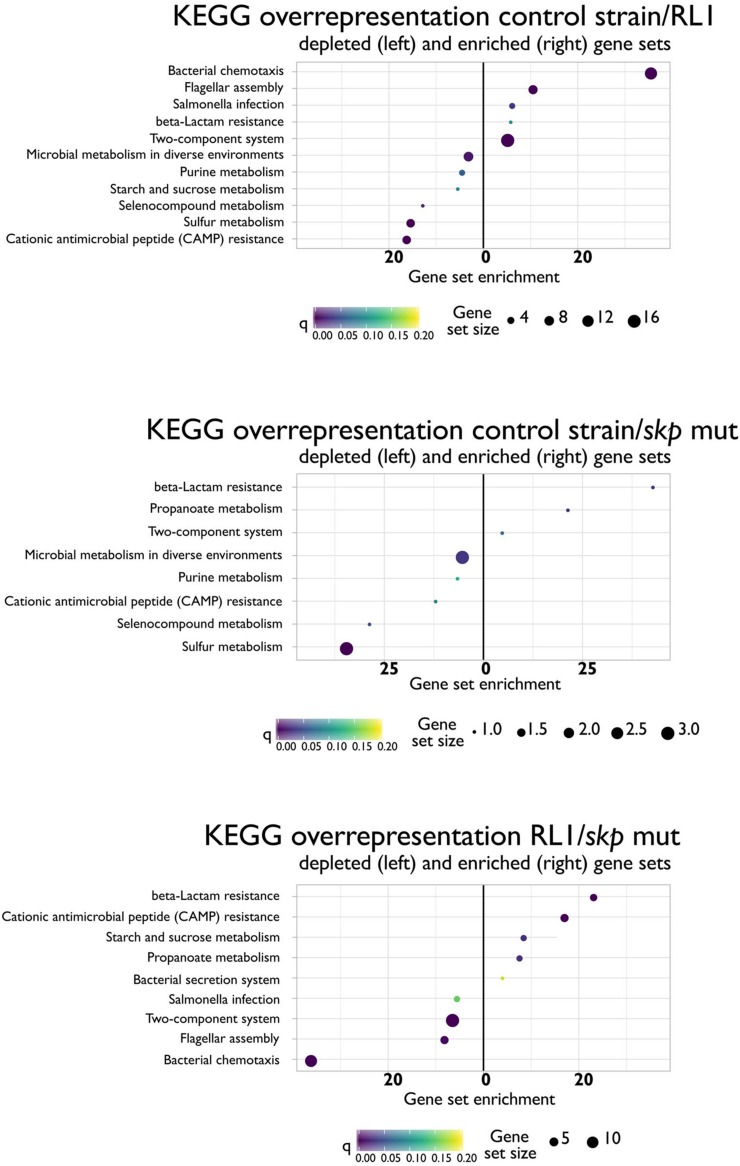
Differences in KEGG cellular pathways between the control strain, RL1, and the *skp* mut. Depletion (left) or enrichment in cellular pathways in the ratio of control strain/RL1 **(upper)**, control strain/*skp* mut **(middle)**, or RL1/*skp* mut **(lower)**. The analysis was performed for mid-log phase bacteria based on three independent replicates for each strain.

In Gram-negative bacteria, 35% of the proteome is directed to the inner and the outer membrane (Mas et al., 2019). Since Skp plays a key role in OMP translocation, we hypothesized that its mutation would have a significant impact on the proteome. Moreover, we aimed to shed light on the resistance mechanism to AMPs of this genotype. We analyzed the proteome of the control strain, RL1, and the *skp* mut strains. Single-protein analysis and pathway analysis, based on the KEGG database, were conducted. First, we found that the Skp protein was expressed in the control strain, but not in RL1 or the *skp* mut bacteria (Supplementary Table S2). This suggests that the mutation led to the loss of this protein. Unlike in the control strain and *skp* mut strains, we did not detect the LPS biosynthesis gene products of *yeiU* and *rfaY* in RL1 (Supplementary Table S2). The RamR protein was found to be expressed similarly among the three strains, suggesting that the *ramR* mutation did not influence protein stability but rather impaired its activity. According to the KEGG database, we found that proteins of the cationic antimicrobial peptide (CAMP) resistance pathway were enriched in RL1 and *skp* mut proteomes compared to the control strain (Figure 7). This was reflected by elevation in HtrA (DegP) both in RL1 and the *skp* mut, compared to the control strain. This protein is known to play a role along with Skp in OMP translocation through the periplasm (Sklar et al., 2007). Additional proteins in the CAMP resistance pathway that were up-regulated in RL1 but not in the *skp* mut are ArnD, YcfS, AcrA, AcrB, and TolC (Supplementary Table S3). The up-regulation of AcrB in RL1 due to the mutation in *ramR* is consistent with our Western blot analysis (Figure 2B) and could also explain the elevation in AcrA levels, as *acrA* and *acrB* are cotranscribed from a single operon (Yamasaki et al., 2013). A depletion in β-lactam resistance pathway was observed in RL1 and *skp* mut (Figure 7), which was enunciated by the reduction of OMPs. A significant reduction in OmpC was observed for *skp* mut, whereas in RL1 additionally OmpF was significantly depleted (Supplementary Table S3). According to the single-gene analysis, we observed a more extensive effect of the *skp* mutation on OMPs. We found a significant reduction of OmpC, OmpD, and OmpF levels both in RL1 and in the *skp* mut compared to the control strain (Supplementary Table S2). However, some OMPs exhibited higher levels in RL1 and *skp* mut compared to control strain. Amounts of OmpX and OmpA were higher in the *skp* mut compared to the control strain, and OmpR and OmpN were found to be more abundant in RL1 compared to the control strain (Supplementary Table S2). Other cellular pathways that were enriched in RL1 and *skp* mut in comparison to the control strain are as follows: microbial metabolism in diverse environments, purine metabolism, selenocompounds metabolism, and sulfur metabolism, which were mostly reflected in up-regulation of the genes in the L-cysteine synthesis pathway (Supplementary Table S3). Compared to the control strain and the *skp* mut, RL1 showed depletion of proteins that are part of the bacterial chemotaxis, flagellar assembly, TCS, and *Salmonella* infection pathways. RL1 showed enrichment in the proteins of the β-lactam resistance, CAMP resistance, and starch and sucrose metabolism pathways (Figure 7). Proteins of the propanoate metabolism pathway were depleted in the *skp* mut compared to the control strain and to RL1 (Figure 7). The enrichment of the bacterial secretion system in RL1 compared to the *skp* mut was reflected by elevation in TolC levels (Supplementary Table S3). To summarize, the *skp* mutation led to the loss of Skp and had a widespread influence on the proteome. Importantly, Skp loss led to a significant reduction in OMPs and to elevation in DegP. Compared to the *skp* mut, RL1 differs more from the control strain in the cellular pathways, apparently, due to additional mutations it carries.

## Discussion

The mutant RL1, isolated after experimental evolution through exposure to the 4DK5L7 AMP, exhibited high level of resistance to this AMP and cross-resistance to others. The resistance is mediated by five spontaneous mutations that were evolved in RL1. Two of the mutations were in LPS biosynthesis genes, *yeiU* and *rfaY*, previously reported to mediate resistance to AMPs by reduction of the surface net negative charge. To our knowledge, the other three mutations that were evolved in RL1 were novel in the context of AMP acquired resistance. We observed that these three mutations mediate resistance to AMPs by two independent mechanisms: one mechanism is drug specific and hence induces hyposensitivity against 4DK5L7 peptide solely, whereas the other mechanism induces hyposensitivity or resistance to multiple AMPs. This strategy of evolving drug-specific and non-specific resistance mechanisms is widely common and was reported previously (Toprak et al., 2011).

Both mechanisms induce only a twofold change in the MIC to the AMP 4DK5L7. These mutations were fixed in the population despite their small contribution, indicating that each conferred a selective advantage. This could be derived from the design of the experimental evolution in which the peptide concentration was increased by merely 50% gradually. The first mechanism involves the AcrAB-TolC system, a multidrug resistance efflux pump that was reported to remove mostly antibiotics, bile salts, and dyes in Gram-negative bacteria (Yu et al., 2003; Pos, 2009) and AMPs in *E*. *coli* (Warner and Levy, 2010) and *K. pneumonia* (Padilla et al., 2009). Here we show that this efflux pump confers resistance to AMPs in *S. typhimurium* as well. The AcrB R620S mutation is within a switch loop, which was found to be important for substrate transport (Eicher et al., 2012). Mutations in several positions in the switch loop (Bohnert et al., 2008; Wehmeier et al., 2009; Cha et al., 2014) and specifically in the R620 position (Ababou and Koronakis, 2016) have been shown to lead to susceptibility to antibiotics. To our knowledge, we are the first to report that mutation within the switch loop induced hyposensitivity toward an AMP. This phenotype was dependent on an additional point mutation T19P in RamR, which led to elevated AcrB levels. Accordingly, the mutation in RamR induced hyposensitivity toward chloramphenicol and ampicillin, well-known substrates of the AcrAB-TolC system (Lim and Nikaido, 2010; Yamamoto et al., 2016). Moreover, we found that the AcrB R620S mutation increased the susceptibility to ampicillin and colistin, indicating that the efflux ability of these two compounds was impaired. RL1 was slightly more susceptible to chloramphenicol and ampicillin compared to the double mutant, although both strains carry mutations in *ramR* and *acrB*. This could be derived from the fact that RL1 carries additional mutations in LPS biosynthesis genes. Lipopolysaccharide serves as a barrier to antibiotics, and alterations in the LPS composition were previously shown to cause susceptibility to antibiotics (Vaara and Nurminen, 1999; Snyder and McIntosh, 2000; Nikaido, 2005; Li et al., 2012). Overall, we suggest that this resistance mechanism involves up-regulation of AcrB through impaired activity of the RamR protein. Thus, elevated levels of mutated AcrB enabled the bacteria to more efficiently extrude the 4DK5L7 peptide. Notably, AcrB substrate sizes vary in the range of several 100 Da. Among the largest substrates reported for the AcrAB-TolC system is bleomycin (Cha et al., 2014). The 4DK5L7 peptide is similar in size to bleomycin, supporting the ability of the efflux pump to transport this AMP.

The second new resistance mechanism is associated with the *hlpA* gene, encoding the Skp periplasmic chaperone. To our knowledge, this gene was never reported to be involved in AMP resistance. We found that the mutation in this gene led eventually to a premature stop codon, thus to the loss of Skp. This induced hyposensitivity to the 4DK5L7 and C8-K5L7 peptides and conferred resistance to seg5D. Interestingly, the resistance to seg5D was more significant than to 4DK5L7, as the MIC was increased fourfold for seg5D compared to twofold for 4DK5L7. This outcome highlights the problem of cross-resistance, which is unexpected and in some cases could be more severe than the resistance to the antibiotic that induced it. Skp, DegP, and SurA are the major three periplasmic chaperones of Gram-negative bacteria that escort OMPs during translocation from the inner to the outer membrane. Skp is a chaperone of the “holdases” family, which prevents substrate aggregation through binding in its cavity, but without directly assisting folding (Walton et al., 2009; Entzminger et al., 2012). DegP is a chaperone/protease with a temperature switch; whereas at low temperatures it has a chaperone activity, in heat shock it has a protease activity (Spiess et al., 1999). In *S. typhimurium*, DegP was found to be important in oxidative stress response and for survival and virulence in mice (Johnson et al., 1991; Humphreys et al., 1999; Mo et al., 2006). The SurA chaperone has a role in OMP folding along with a peptidyl-prolyl isomerase activity (Goemans et al., 2014). To date, the exact contribution of each chaperone to OMP biogenesis is still controversial. According to one model, Skp/DegP and SurA are two parallel redundant pathways for OMP translocation through the periplasm (Rizzitello et al., 2001). Another model suggests that Skp/DegP rescues OMPs that fall off SurA (Sklar et al., 2007). Alternatively, it was suggested that Skp and SurA act sequentially in the same pathway (Bos and Tommassen, 2004). Nevertheless, previous reports demonstrated that Skp depletion in *E. coli* had a negligible impact on OMP levels (Sklar et al., 2007; Denoncin et al., 2012). In contrast, we observed that the loss of Skp led to a significant reduction in major OMPs including OmpC, OmpF, and OmpD. It is possible that the role of Skp in *S. typhimurium* is more essential than in *E. coli*. A similar phenotype was observed in *Neisseria meningitidis*, where Skp depletion resulted in a severe reduction in the major OMPs (Volokhina et al., 2011). Misfolded OMP stress is detected by the CpxA/R TCS or by the alternative sigma factor σ^E^, both of which up-regulate the transcription of DegP (Masi et al., 2019). Accordingly, we found that RL1 and *skp* mut expressed higher levels of DegP compared to the control strain. It was previously shown that DegP has a relation to the CAMP resistance pathway, possibly through assisting the assembly of transporters that mediate efflux of AMPs (Weatherspoon-Griffin et al., 2014) or by proteolytic degradation (Ulvatne et al., 2002). It is possible that resistance to AMPs in the *skp* mut is derived from the up-regulation of DegP. However, it is less likely that the resistance mechanism involves proteolytic degradation as both 4DK5L7 and the seg5D contain D amino acids, which were shown to protect them against proteolytic degradation (Papo et al., 2002; Papo and Shai, 2005). Therefore, it is possible that DegP functions in the support of biogenesis of membrane-associated multidrug transporters that mediate resistance to those AMPs. Nonetheless, we cannot rule out that other cellular pathways that were affected both in RL1 and the *skp* mut or the alterations in bacterial envelope due to the periplasmic stress confer resistance to the studied AMPs.

## Conclusion

Antimicrobial peptides resistance can be a complex multimechanistic phenotype involving multiple genes. Here we report on a multimechanism resistance that involves mutations in five genes; three of the mutations are associated with AMP acquired resistance for the first time. Our data reveal two parallel resistance mechanisms that were evolved in RL1. The first mechanism involves two mutations, which resulted in overexpression of a mutated AcrB protein, which together improved the efflux of 4DK5L7. However, this resistance mechanism had a cost, as bacteria became hypersusceptible to ampicillin and colistin. The second mechanism induced hyposensitivity to multiple AMPs and was achieved by a mutation in *skp*, which abolished this protein. This led to a reduction in OMPs and to elevation in DegP and influenced multiple cellular pathways. Further investigations are required to confirm the molecular mechanism conferring resistance to AMPs by the *skp* mutation.

## Data Availability Statement

The genome sequencing data have been deposited to BioProject accession number PRJNA545195 in the NCBI BioProject database. The mass spectrometry proteomics data have been deposited to the ProteomeXchange Consortium via the PRIDE (Perez-Riverol et al., 2019) partner repository with the dataset identifier PXD014818.

## Author Contributions

GK and RN contributed conception and design of the study. GK, RN, AD, and SF performed the experiments. CS generated the mutants. AS performed the proteomic analysis. GK and RG analyzed the data. GK wrote the first draft of the manuscript. RN, RG, and YS revised the manuscript. YS and RG supervised this project.

## Conflict of Interest

The authors declare that the research was conducted in the absence of any commercial or financial relationships that could be construed as a potential conflict of interest.

## Footnotes

1 imagej.nih.gov/ij/

2 https://www.r-project.org/

## Supplementary Material

FIGURE S1 HA mutants with 4DK5L7. Growth curves of the bacteria with the 4DK5L7 peptide at **(A)** 25 μM, **(B)** 12.5 μM, **(C)** 6.25 μM, and **(D)** without peptide. OD_600_ was monitored every 20 min for 18 h. The values are average of at least three biological replicates, each performed in duplicates. Error bars indicate the standard error. Statistical analysis was performed using *Tukey multiple comparison test* for the last five the time points. Statistically significant differences are indicated as a≠b≠c, *P* < 0.05.

TABLE S1 Primers that were used in this study.

TABLE S2 Description of the genes that were depleted or enriched according to the single gene analysis.

TABLE S3 Description of the genes that were depleted or enriched according to the KEGG pathways database analysis.
