# Supplementary figures and images for: Loss of the Periplasmic Chaperone Skp and Mutations in the Efflux Pump AcrAB-TolC Play a Role in Acquired Resistance to Antimicrobial Peptides in Salmonella typhimurium
Source: Front Microbiol. 2020 Mar 10;11:189. doi: 10.3389/fmicb.2020.00189 (PMC7075815; doi:10.3389/fmicb.2020.00189)

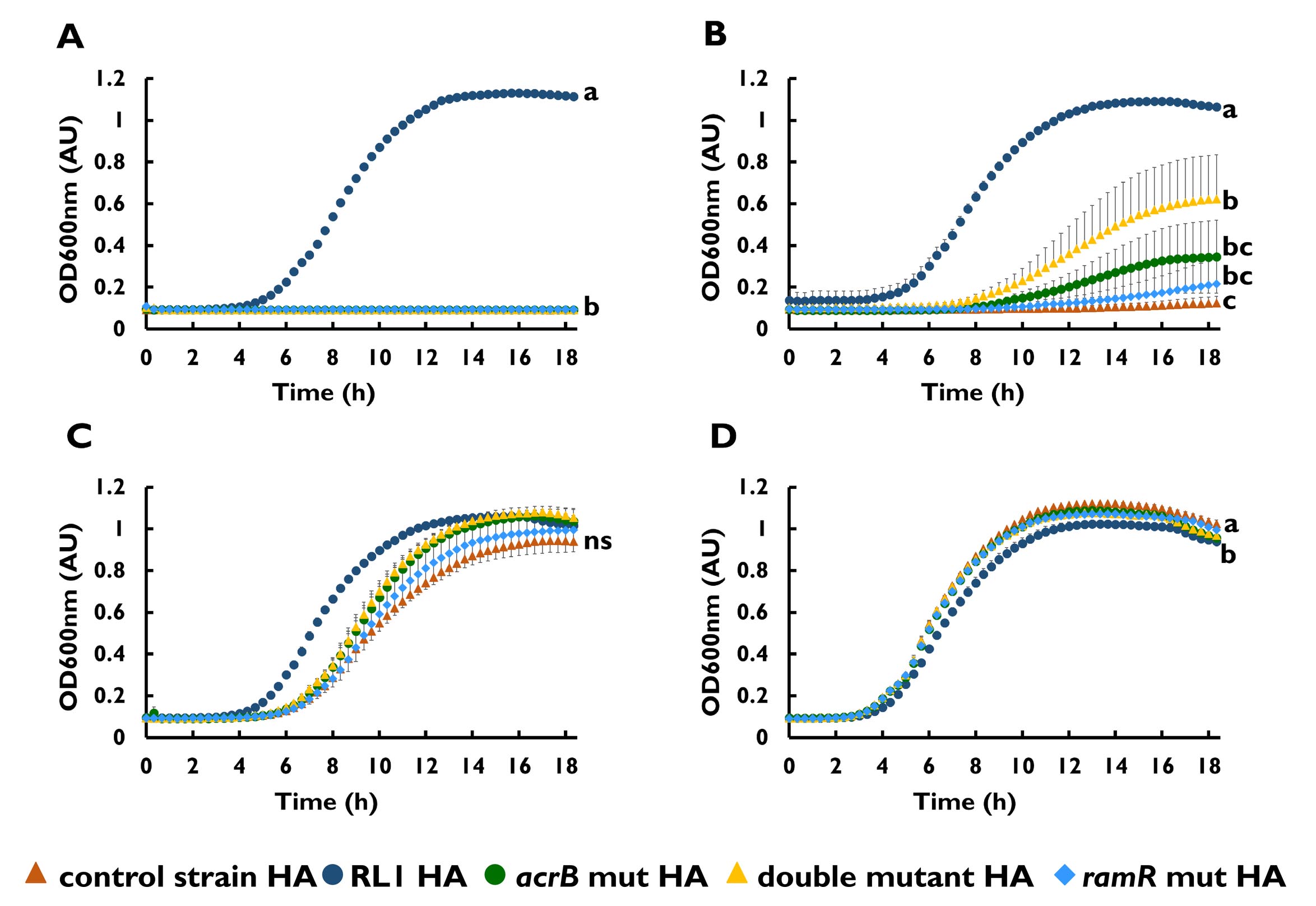

Supplement: FIGURE S1 — HA mutants with 4DK5L7. Growth curves of the bacteria with the 4DK5L7 peptide at (A) 25 μM, (B) 12.5 μM, (C) 6.25 μM, and (D) without peptide. OD600 was monitored every 20 min for 18 h. The values are average of at least three biological replicates, each performed in duplicates. Error bars indicate the standard error. Statistical analysis was performed using Tukey multiple comparison test for the last five the time points. Statistically significant differences are indicated as a≠b≠c, P < 0.05. [file Image_1.JPEG]
